# Medical students and psychiatry: an attitude change?

**DOI:** 10.3402/meo.v18i0.20676

**Published:** 2013-04-02

**Authors:** Rashi Aggarwal, Nicole Guanci, Giovanni Caracci, Erika Concepcion

**Affiliations:** 1 Department of Psychiatry, UMDNJ–New Jersey Medical School, Newark, NJ, USA; 2 Department of Psychiatry, Beth Israel Medical Center, New York, NY, USA

Medical student attitudes toward psychiatry form the foundation of their future interactions with psychiatric patients and their decision-making process regarding pursuing psychiatry as a career (1). Related studies have identified positive views overall, but some degree of stigma against psychiatry still persists among students (2–5). Psychiatric educators have been aware of this finding for decades and have considered exposure to psychiatry the principal factor influencing medical student attitudes toward the field (4–7). However, medical students’ opinions are formed by exposure to patients during their psychiatric clerkships and also by their experience on other clerkships, particularly when treating patients with comorbid psychiatric problems and observing the attitudes of non-psychiatric staff toward psychiatry (8).

We looked at medical student attitudes at our own institution, the University of Medicine and Dentistry of New Jersey – New Jersey Medical School. The questionnaire used with permission was designed by Balon et al. and consists of 23 questions assessing the perceived merits of psychiatry, efficacy, role definition and functioning, possible abuse, and career and personal reward (2).The students surveyed consisted of first-year (*N*=166, response rate 92%) and fourth-year students (*N*=110, response rate = 61%). Our students’ attitudes were generally consistent with other studies, revealing a positive view of psychiatry overall. In fact, most (91% either strongly or moderately agreed) students believed that psychiatric research has made good strides in advancing care of the major mental disorders. In addition, most (88% either strongly or moderately) agreed that psychiatric consultation in a clinical setting is fruitful and that treatment is most often helpful to most people who receive it (81%).

Interestingly, comparisons between first- and fourth-year medical students showed no statistically significant differences in any of the 23 questions compared. These results were in contrast to our expectation to find more significant differences between first- and fourth-year student opinions, particularly since first-year medical students were assessed at the onset of their formal medical education and fourth-year students after completing their core clerkships, including psychiatry. It is possible that by observing relapses and readmissions among patients with chronic psychiatric illnesses, students felt discouraged about the value of psychiatric treatment. Also, it is plausible that the fourth-year students’ impressions of psychiatry were negatively impacted by medical professionals in other specialties – a consideration consistent with a study by Ndetei et al. in Kenya, which found that first years with no exposure to psychiatry had the most positive attitude toward psychiatry compared to other students (3).

These findings led us to consider the role medical education plays in changing the attitudes of students toward psychiatry. Can medical education impact all aspects of student views or are some views reflective of societal opinions against psychiatry, and, therefore, less modifiable by medical school experience? It may be presumed that medical students would be better informed, and thus, less biased than the general populace. However, some authors hypothesize that medical school selection criterion are inherently prejudiced against students interested in psychiatry (9). Studies have found that students with backgrounds in humanities or social sciences are more likely to choose psychiatry as a career (10). However, students entering medical school tend to focus on the sciences, as entrance criteria are more heavily weighted toward MCAT scores and GPA than toward interpersonal skills and empathy.

Regardless, it appears that stigma against psychiatry remain despite efforts by educators and advances in the field of psychiatry. Future studies should focus on the full experience of medical school as an influencing factor rather than just the psychiatry clerkship. Also, studies must distinguish between factors that medical schooling can modify versus factors that reflect more deeply ingrained beliefs reflective of societal perceptions. The latter may be addressable by medical school admission policy changes.
